# Measurement of Spiritual Wellbeing in an Australian Hospital Population Using the Functional Assessment of Chronic Illness Therapy: Spiritual Wellbeing Scale (FACIT-Sp-12)

**DOI:** 10.1007/s10943-024-02064-x

**Published:** 2024-06-13

**Authors:** Megan C. Best, Grahame Simpson, Kate F. Jones, Frankie Merritt, Michael Casey, Sandra Lynch, John A. Eisman, Jeffrey Cohen, Darryl Mackie, Kirsty Beilharz, Matthew Kearney

**Affiliations:** 1 Institute for Ethics and Society, University of Notre Dame Australia, PO Box 944, Broadway, Sydney, NSW 2007 Australia; 2 Faculty of Medicine and Health, The University of Sydney, Sydney, Australia; 3 John Walsh Centre for Rehabilitation Research, Northern Sydney Local Health District, Sydney, Australia; 4 School of Medicine, Sydney, University of Notre Dame Australia, Sydney, Australia; 5 School of Philosophy and Theology, The University of Notre Dame Australia, Australia Sydney,; 6 IMPACCT Research Centre, Faculty of Health, University of Technology, Sydney, Australia; 7 Garvan Institute of Medical Research, Darlinghurst, Australia; 8 School of Medicine, University of New South Wales, Sydney, Australia; 9 St Vincent’s Hospital, Sydney, Australia; 10 St. Vincent’s Private Hospital, Sydney, Australia; 11 School of Arts and Sciences, The University of Notre Dame Australia, Sydney, Australia

**Keywords:** Spirituality, FACIT-Sp-12, Spiritual wellbeing, Hospital, Patients

## Abstract

Spiritual wellbeing is known to be a predictor of increased patient coping in hospital settings. Therefore, access to a valid and reliable measure of spiritual wellbeing amongst general hospital patients is highly recommended. The aim of this study was to investigate the dimensionality, reliability, and validity of the Functional Assessment of Chronic Illness Therapy Spiritual Wellbeing scale (FACIT-Sp-12) in a heterogeneous cohort of hospital patients. A cross-sectional survey was administered to 897 adult patients across six hospitals in Sydney, Australia. Confirmatory factor analysis for the three-factor FACIT-12-Sp indicated a poor fit, but after removal of Item 12, the three-factor FACIT-11-Sp presented a good fit to the data. Reliability testing indicated acceptable to good internal consistency. Validity was supported by statistically significant differences between patients who considered themselves ‘both spiritual and religious’ and ‘not religious or spiritual’. While some caution should be taken when using the FACIT-Sp due to several limitations, nevertheless, in a general hospital population in Australia, the three-factor FACIT-11-Sp indicated good dimensionality, reliability, and validity.

## Introduction

Spirituality and its relationship to health outcomes is a growing field of research. High levels of spiritual wellbeing have been associated with increased coping and resilience with illness and hospitalisation, including in those with a very broad range of general medical illnesses, including chronic pain, kidney disease, diabetes, pulmonary disease, cancer, blood disorders, cardiovascular disease, dental or vision problems, neurological disorders, HIV/AIDS, systemic lupus erythematosus, bowel disease, musculoskeletal disease, psychiatric illness, as well as end of life issues (Koenig et al., 2023; Koenig et al., 2023). High spiritual wellbeing has indeed been suggested to be as important as physical wellbeing for quality of life in cancer patients (Brady et al., 1999).

A review of the literature reported that spirituality is important to most patients with serious illness, with spiritual needs common in that setting, and spiritual care desired by the majority of patients (Balboni et al., 2022). Spirituality can also have a negative impact on patient outcomes as it can impact medical decision making, potentially providing a barrier to compliance with treatment (Balboni et al., 2022; Padela et al., 2012; Pargament et al., 2004). Assessment of patient spiritual wellbeing would therefore be an important step in ensuring that holistic care is provided in the hospital setting.

Access to a valid and reliable measure of spiritual wellbeing amongst hospital patients is therefore highly desirable. However, a review of questionnaires measuring dimensions of spirituality and religiosity in clinical settings found that most were validated in oncological and palliative care settings (Austin et al., 2018). Of these, the FACIT-Sp-12 is the most frequently used.

### The FACIT-Sp-12 Spiritual Well-Being Scale

The FACIT-Sp-12 is a validated measure of spiritual wellbeing that was developed within a broad conceptualisation of spirituality, described as ‘a personal search for meaning and purpose in life, connection with a transcendent dimension of existence, and the experiences and feelings associated with that search and that connection’ (Peterman et al., 2002), rather than as specific religious beliefs and practises. It was designed to be used with patients experiencing chronic and life-threatening conditions to measure the level of peacefulness, meaning and purpose, and faith of medical patients.

Empirical research has demonstrated that the FACIT-Sp-12 is a psychometrically sound measure of spiritual wellbeing. The original scale comprised two subscales: meaning/peace and faith (Peterman et al., 2002), however, more recent studies have reported a three-factor model as a better fit: meaning, peace and faith (Canada et al., 2008; Whitford & Olver, 2012). Thus, there remain questions about its specific factor structure and the validity of the scores from its separate scales, including whether the subscales of meaning and peace are distinct and hence reinforce the proposed three factor structure (Peterman et al., 2014). However, Park and colleagues welcomed the introduction of the three factor model as providing more informative constructions and better psychometric properties in view of the close relationship between the meaning/peace component and anxiety (Park et al., 2017).

The FACIT-Sp-12 has been translated and linguistically validated in 39 languages. It consists of 12 items rated on a 5-point Likert scale (0 = Not at all–5 = Very much). Possible spiritual wellbeing scores range from 0 to 48, with higher scores reflecting greater spiritual wellbeing. The recall period for the questions is 7 days (Peterman et al., 2002).

### Spiritual Care in Australia

Australia is a culturally diverse nation, with the most recent national census figures revealing a trend away from formal religion, given the number of Australians identifying with ‘no religion’ increased from 19% in 2006 to 30% in 2016 and 38% in 2021. At the same time, religious diversity has increased, with Hinduism and Islam the fastest growing religions, although Christianity remains the most common (43.9%) (Australian Bureau of Statistics, 2021). The importance of spiritual care has been recognised at a policy level in Australia, with national organisations mandating accessibility of safe and high-quality spiritual care in healthcare and aged care in view of its importance to quality of life and well-being (Meaningful Ageing Australia, 2016; Spiritual Health Association, 2020). However, if the impact of spiritual care is to be assessed, it is necessary to identify a reliable measure of spiritual wellbeing.

We looked at the FACIT-Sp-12 in a diagnostically and demographically heterogeneous Australian medical population, aiming to examine how it performed in the context of a general hospital admission. We predicted that patients who considered themselves ‘spiritual but not religious’ or ‘both spiritual and religious’ would have significantly higher FACIT-Sp-12 scores than the patients who aligned themselves with the ‘Neither religious nor spiritual’ group. This is the third paper from a larger study which illustrated that admission to hospital is a significant life event, which can be challenging even if not life threatening, and may be associated with spiritual need (Best et al., 2022, 2023).

## Methods

This was a cross-sectional study comprising a short survey.

### Participants

Participants were recruited from six hospitals across Sydney, Australia. Study hospitals included three public, two private, and one combined public/private facilities, comprising both acute and sub-acute inpatient as well as outpatient care, and represented a combined total of over 1000 beds. Hospitals included both faith-based and non-faith-based institutions. Eligible patients were adult; alert, oriented and able to give verbal consent; able to understand and speak English; and well enough to participate in the study. Healthy women admitted to maternity units were excluded on grounds of the absence of pathology.

Eligible patients were identified by nursing unit managers at the participation sites and approached by a researcher, who asked whether they were willing to participate in a short survey, explained what it involved and answered any questions. Efforts were made to identify a heterogeneous sample reflecting the demographic composition of the population being studied. Verbal consent was obtained before the survey was distributed and documented by the return of an anonymous survey (implied consent). Ethics approval was granted by the Human Research Ethics Committee at St Vincent’s Hospital, Sydney (HREA AU/1/B78D25).

### Procedure

All participants were asked to complete a paper questionnaire which included the following: Demographic details, self-assessment of spirituality and religiosity (Fetzer Institute and National Institute on Ageing, 1999), and the FACIT-Sp-12 (Peterman et al., 2002). Racial characteristics were not collected in this cohort. Patients unable to write were assisted by the researcher. Recommended sample size for this project was proposed at *n* = 500–1000 (Boateng et al., 2018).

### Statistical Analysis

Data were entered into the Statistical Package for the Social Sciences (SPSS) for Windows version 24 (SPSS Inc., Chicago, IL, USA, 2021) and descriptive statistics calculated (means, standard deviation). Three psychometric aspects of the FACIT-Sp-12 were investigated in the current report, namely dimensionality, reliability and validity, consistent with the guidelines recommend by Koenig and Zaben (2021). Some minor differences in sample numbers are noted across the three sets of analyses due to small amounts of missing data.

Dimensionality was tested by Confirmatory Factor Analysis (CFA) to confirm the *a priori* factor structure of the FACIT-Sp-12. One form of reliability, namely Cronbach’s alpha, was then undertaken, to test the internal consistency of scale items, applying the interpretation proposed by George and Mallery (2019):  ≥ 0.9—Excellent, ≥ 0.8—Good, ≥ 0.7—Acceptable, ≥ 0.6—Questionable, ≥ 0.5—Poor, and ≤ 0.5—Unacceptable (p. 231).

Finally, validity was tested through differentiation amongst known groups and undertaken in two steps. Analyses employed a one-way Analysis of Variance (ANOVA), with post-hoc Sheffé tests conducted if the global F statistic was significant. First, differences were tested based on patient group, with the prediction that there would be no difference amongst groups as type of patient (e.g., Medical, Surgical, etc.) has no explicit relationship to level of spirituality or religiosity or severity of illness.

Second, differences were tested employing responses from the self-assessment of spirituality and religiosity. Respondents were organised into five groups, namely ‘Neither religious nor spiritual’ (NRS), ‘Spiritual but not religious’ (SNR), and ‘Both spiritual and religious’ (BSR), ‘Religious but not spiritual’ (RNS) and ‘Neither agree nor disagree’ (NAD). The last two groups were not included in the analysis due to small numbers (*N* = 20 and *N* = 40 respectively).

## Results

Responses were received from 897 patients. There were more males than females, with more than half the sample aged over 60 years old, reflecting the sample population. A range of diagnostic groups were represented. Almost 65% identified as Roman Catholic, Orthodox or Protestant Christian with over 25% not identifying with any religious affiliation, reflecting a more religious cohort than the Australian average. When asked whether they considered themselves to be spiritual or religious, over one third described themselves as spiritual and religious, and almost one third as neither spiritual nor religious. See Table 1 for demographic, patient group, and spirituality details.

**Table 1:** Participant demographic characteristics *n* = 897

Demographic items	Category	*N* (%)
Gender	Female	389 (45.0)
	Male	469 (54.2)
	Missing	7 (0.8)
Age (*n*, %)	20–29	58 (6.7)
	30–39	33 (3.8)
	40–49	56 (6.5)
	50–59	115 (13.3)
	60–69	173 (20.0)
	70–79	224 (25.9)
	80 and over	126 (14.6)
	Missing	80 (9.2)
Type of patient group (*n*, %)	Medical	338 (39.1)
	Surgical	232 (26.8)
	Rehabilitation	84 (9.7)
	Emergency Medicine	64 (7.4)
	Palliative care	55 (6.4)
	Geriatric/Aged Care	35 (4.0)
	ICU	21 (2.4)
	Psychiatry	10 (1.2)
	Other	21 (2.4)
	Missing	5 (0.6)
Religious affiliation (*n*, %)	Protestant	316 (36.5)
	Roman Catholic/Orthodox	242 (28.0)
	None	229 (26.5)
	Jewish	35 (4.0)
	Other religions*	25 (2.9)
	Other	12 (1.6)
	Missing	6 (0.7)
I am a religious or spiritual person**	Neither spiritual nor religious	259 (29.9)
	Spiritual but not religious	142 (16.4)
	Spiritual and religious	307 (35.5)
	Neither agree nor disagree	40 (4.6)
	Religious but not spiritual	20 (2.3)
	Missing	97 (11.2)

*Other religions: Islam (*n* = 13), Hindu (*n* = 7), Buddhist (*n* = 3), Indigenous spirituality (*n* = 2). **Neither spiritual or religious: Not spiritual (disagree or strongly disagree) and not religious (disagree or strongly disagree); Spiritual but not religious: Spiritual (Strongly agree or agree) but not religious (disagree or strongly disagree); Both spiritual and religious: Spiritual (Strongly agree or agree) and religious (Strongly agree or agree); Neither agree nor disagree (neither agree/disagree for both); Religious but not spiritual: Religious (Strongly agree or agree) but not spiritual (strongly disagree or disagree)

### Dimensionality

**Fig. 1:**
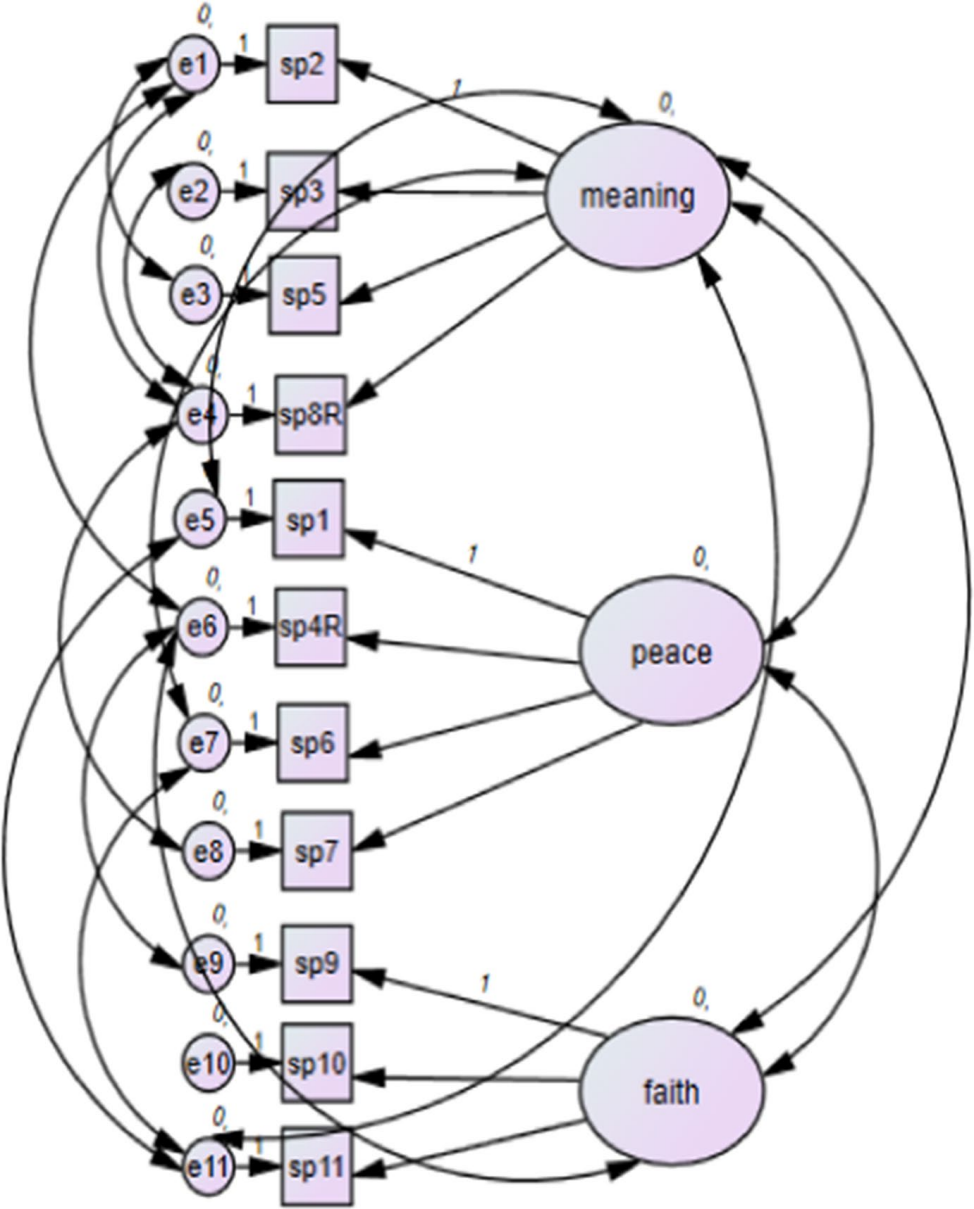
Three-factor FACIT-11 Sp model including modification indices

Confirmatory factor analysis (CFA) including the use of modification indices, was conducted on the three-factor FACIT-12-Sp model: peace (sp1, sp4R, sp6, sp7); meaning (sp2, sp3, sp5, sp8R); faith (sp9, sp10, sp11, sp12). Baseline comparisons and the RMSEA were used to determine the fit of the model to the data. The baseline comparisons were all < 0.90 and RMSEA > 0.80, indicating a poor fit. Item 12 (‘I know that whatever happens with my illness, things will be okay’) was removed due to low inter-item correlations. CFA was then repeated on the three factor FACIT-11-Sp model with item 12 removed. This three-factor model presented a good fit to the data (NFI = 0.959, RFI = 0.922, IFI = 0.965, TLI = 0.934, CFI = 0.965 and RMSEA = 0.75). See Fig. 1.

### Reliability

For both the reliability and validity testing below, the FACIT-Sp-12 was tested alongside the 11-item version indicated by the CFA. Reliability testing on the total score indicated Acceptable to Good internal consistency of the FACIT-Sp-11 and FACIT-Sp-12 across most age categories, gender, and the total group (see Table 2). The internal consistency coefficients for the Faith subscale also fell into the Acceptable range (with or without item 12). However, the Meaning and Peace subscale scores had poorer internal consistency, rated at Poor and Questionable, respectively. The reliability co-efficient for the combined Meaning and Peace subscales approached Acceptable and was stronger than the coefficients for the two individual subscales.

**Table 2:** Cronbach alphas

	*N* (%)	*M* (SD)	Cronbach alpha
Total group (FACIT-SP 12)	862	32.3 (8.9)	0.79
Total group (FACIT-SP 11)	862	29.0 (8.2)	0.76
Meaning (2,3,5,8R)	865	12.9 (2.7)	0.55
Peace (1, 4R, 6,7)	862	10.4 (4.0)	0.64
Meaning and Peace (Items 1–8)	862	23.3 (5.6)	0.68
Faith (9, 10, 11, 12)	865 (100.0)	9.0 (4.7)	0.76
Faith (9, 10, 11)	865 (100.00)	5.7 (4.1)	0.79
FACIT-SP 12 by age and gender			
Age category (*N* = 782)			
20–29	57 (6.5)	27.8 (10.0)	0.85
30–39	32 (4.1)	32.2 (9.9)	0.83
40–49	56 (7.2)	33.8 (8.9)	0.81
50–59	115 (14.7)	33.2 (9.4)	0.82
60–69	173 (22.1)	33.1 (8.7)	0.77
70–79	223 (28.5)	32.6 (7.6)	0.71
80 and older	126 (16.1)	32.1 (9.0)	0.79
Gender (*N* = 855)			
Males	466 (54.5)	32.4 (9.1)	0.8
Females	389 (45.5)	32.4 (8.7)	0.77
FACIT-SP 11 by age and gender			
Age category (*N* = 782)			
20–29	57 (6.5)	25.1 (9.0)	0.83
30–39	32 (4.1)	28.7 (9.3)	0.81
40–49	56 (7.2)	30.4 (8.4)	0.8
50–59	115 (14.7)	29.8 (8.6)	0.8
60–69	173 (22.1)	29.8 (8.1)	0.75
70–79	223 (28.5)	29.2 (7.0)	0.67
80 and older	126 (16.1)	29.0 (8.1)	0.76
Gender (*N* = 855)			
Males	466 (54.5)	29.1 (8.3)	0.77
Females	389 (45.5)	29.1 (8.0)	0.75

### Validity

Analysis found significant difference based on known groups. As predicted, there were no statistically significant differences between patient group means on the FACIT-Sp-11 (*F*(5,802) = 0.72, *p* = 0.610) or FACIT-Sp-12 (*F*(5,800) = 0.55, *p* = 0.741) as determined by one-way ANOVA (see Table 3).

**Table 3:** FACIT-SP scores by patient group

	*N* (%)	FACIT-SP 12	FACIT-SP 11
		*M* (SD)	*M* (SD)
Medical	338	32.4 (8.9)	29.2 (8.1)
Surgical	230	32.3 (9.0)	29.0 (8.3)
Rehabilitation	84	31.0 (9.5)	27.7 (8.7)
Emergency medicine	64	32.8 (7.7)	29.5 (6.8)
Palliative care	55	31.4 (9.5)	28.0 (8.9)
Geriatric/aged care	35	33.0 (9.0)	29.9 (7.9)

However, there was a statistically significant difference on FACIT-Sp-11 (*F*(2,705) = 20.31, *p* < 0.001) and FACIT-Sp-12 (*F*(2,703) = 18.18, *p* = 0.000) scores according to how religious or spiritual a person rated themselves, once again as predicted (see Tables 4, 5). Post-hoc (Scheffé) tests showed a significant difference between groups of participants who identified as ‘Not religious or spiritual’ and ‘Both spiritual and religious’, and ‘Not religious or spiritual’ and ‘Spiritual but not religious’. There was no significant difference between ‘Both spiritual and religious’ and ‘Spiritual but not religious’ groups. No significant difference on FACIT-Sp-12 scores was recorded between different religious affiliations.

**Table 4:** FACIT-SP scores by spiritual/religious category (*n* = 708)

	*N*	FACIT-SP 11	FACIT-SP 12
		*M* (SD)	*M* (SD)
Neither spiritual nor religious	259	26.9 (8.3)	30.13 (9.1)
Spiritual but not religious	142	29.5 (8.0)	32.66 (8.8)
Both spiritual and religious	307	31.2 (7.8)	34.62 (8.5)

**Table 5:** Comparison of FACIT-SP scores between groups

Test	FACIT-SP 11 *p* value	FACIT-SP 12 *p* value
NRS versus SNR	0.01	0.023
NRS versus BSR	< 0.001	< 0.001
SNR versus BSR	0.098	0.092

NRS, Neither spiritual nor religious: Not spiritual (disagree or strongly disagree) and not religious (disagree or strongly disagree); SNR, Spiritual but not religious: Spiritual (Strongly agree or agree) but not religious (disagree or strongly disagree); BSR, Both spiritual and religious: Spiritual (Strongly agree or agree) and religious (Strongly agree or agree)

## Discussion

This study sought to validate the FACIT-Sp-12 in a heterogeneous cohort of Australian hospital patients. The FACIT-Sp-12 was developed with a sample of cancer patients in the USA (Peterman et al., 2002). In our study we calculated a median score of 32.4 across the whole sample. This finding is similar to studies of caregivers of palliative care patients in Australia, where the median score was 30.5 (O’Callaghan et al., 2020).

A cohort of American cancer survivors who were assessed to establish reference values for the FACIT-Sp-12 found significantly higher scores in women (the reference group) (37.84), older adults (38.11) and Black non-Hispanics (39.95) (Munoz et al., 2015). We found higher scores in older adults, but no difference between sexes. Whitford and colleagues, in an Australian sample of cancer patients, found that scores were higher in those with religious affiliation and for whom religion was important (34.0–34.4), a trend which was also found in our sample (Whitford & Olver, 2012).

We found on CFA that the measure performed more strongly when Item 12 was removed. Item 12 is a measure in the faith subscale, ‘I know that whatever happens with my illness, things will be okay.’ We are not sure whether this was a result of a weakness in the scale design, or a reflection of the characteristics of this Australian cohort which was more medically heterogenous than the original target group. Item 12 is distinct from the other questions in the Faith subscale: (9) ‘I find comfort in my faith or spiritual beliefs’; (10) ‘I find strength in my faith or spiritual beliefs’; and (11) ‘My illness has strengthened my faith or spiritual beliefs’. Item 12 does not mention faith/spirituality, and also focuses on the future, rather than the present.

Koenig (2012) suggests that the peacefulness, purpose, strength and comfort investigated by the scale are the results of living a spiritual life rather than spirituality itself. He notes that the FACIT-Sp’s meaning and peace subscales especially are a measure of positive mental health and that Item 12 is a measure of optimism. The measure is therefore contaminated and acts as a marker of good mental health (i.e., successful coping) (page 223), rather than spiritual wellbeing alone.

The commonality between metaphysical constructs such as meaning of life and psychological determinants is not surprising but highlights the complexity of research in this area. Other authors have identified these limitations with regard to the FACIT-Sp (Deng et al., 2022; Olver & Whitford, 2014; Öztürk et al., 2023; Park et al., 2017). In their study of patients with heart failure, while Deng and colleagues found changes in spiritual wellbeing over time, they noted the conceptual overlap between items in the FACIT-Sp and general constructs of wellbeing which could have accounted for some of their findings (Deng et al., 2022). In their study of resilience in Chronic Obstructive Pulmonary Disease patients, Öztürk and colleagues noted that, since FACIT-Sp evaluates the concept of spirituality through positive mental states such as the meaning of life, finding purpose, and feeling peaceful, it is an expected result that the scale will be associated with positive emotions such as resilience (Öztürk et al., 2023). Park et al. (2017) note that care should be taken to avoid using the FACIT-Sp as a predictor of wellbeing outcomes, a common misuse that confounds predictor and outcome variables.

These findings suggests that use of the FACIT-Sp should be limited to assessment in the clinical context to measure psychological wellbeing and use of faith. It should not be used when conducting research examining the relationship between spirituality and mental health where precise separation of concepts is required. Instead, scales that measure religious involvement and are not contaminated with indicators of positive mental health can be used, such as the Duke University Religion Index (DUREL) (Koenig & Büssing, 2010), the Religious Commitment Inventory (Worthington Jr et al., 2003), the Intrinsic Religiosity Scale (Hoge, 1972), or the Daily Spiritual Experiences Scale (either dropping the indicators of peacefulness and social connection with others, or administering the entire scale and then analysing the results with and without those positive mental/social health indicators) (Underwood, 2011). Of these, Koenig suggests that, while the measure used should be contextualised to the individual project, Hoge’s Intrinsic Religiosity Scale is the best overall (Koenig, 2012).

This raises the question of how best to measure spiritual wellbeing in populations with low religiosity if contamination is to be avoided, especially as a more holistic understanding of care does not compartmentalise psychology and spirituality. Ensuring content validity for measures of spiritual wellbeing will be of great importance and more research is needed to identify how to measure spiritual wellbeing in populations with low religiosity and/or where a broader definition of spirituality, such as the popular ‘consensus definition’ (Puchalski et al., 2014) is used. Büssing has suggested that, in secular research contexts, it is useful to measure spirituality and religiosity separately. The Awe/Gratitude questionnaire (GrAw-7) (Büssing et al., 2018) which measures transcendent feelings associated with non-religious spiritual experience and is not contaminated with specific religious topics or quality of life constructs, shows promise in this area.

We found that mean responses to the FACIT-Sp Item number 12 are, on average, more positive than those of the other 11. Therefore, for those wanting to compare the 11-item to 12-item, it appears that the prorating could underestimate the actual 12-item score. Therefore, we recommend that such future studies administer all 12 items and score both ways. While we have found the 11-item version to be more robust, we have provided the 12-item scores here to enable comparison with other studies. However, in view of our findings, we would only recommend use of the FACIT-Sp-11 for use by chaplains and healthcare professionals to assess level of peacefulness, meaning and purpose and to some extent the faith of medical patients from a clinical standpoint only.

This cohort previously reported that spiritual needs fluctuated during hospitalisation and that increased needs may not be anticipated in advance (Best et al., 2023). The situation is different for patients diagnosed with cancer and chronic disease (the cohort in whom this measure was developed), for whom problems in the future are guaranteed. Although over a quarter (26.5%) of our cohort had no religious affiliation, over half (51.9%) considered themselves spiritual. Despite declining religiosity in Australia, data indicates that spiritual beliefs remain important in providing meaning during times of stress, such as serious illness and hospitalisation (Best et al., 2022). The importance of spirituality in serious illness has been identified internationally (Balboni et al., 2022).

The study’s research question was twofold, namely that participants who identified as ‘Both spiritual and religious’, or ‘Spiritual but not religious’ would score more highly for spiritual wellbeing than those who identified as ‘Not religious or spiritual.’ We found that this was the case, with statistically significant differences between the spiritual and non-spiritual groups. This finding has implications for the way spiritual care is provided in inpatient settings.

Current practice in Australia involves asking for religious affiliation at the time of hospital admission (a practice that may be omitted), and sometimes a question regarding whether the patient would like to be seen by a chaplain (also known as a spiritual care practitioner or pastoral care provider). While some hospitals with sufficient staff endeavour to provide spiritual care to all inpatients who do not object, the underlying assumption in Australia is that spiritual care is only required by patients who identify as religious and those who request it. However, if spiritual care provision is limited to those with a religious affiliation or who proactively request spiritual care, many patients who might potentially benefit from spiritual care may be overlooked in the allocation of services. This finding has been identified in other studies (Balboni et al., 2022).

Rather than relying on patient self-identification at the time of admission, the standardised use of the generalist-specialist model of spiritual care provision embedded within clinical teams (Handzo & Koenig, 2004) would ensure that those patients who require spiritual care would receive it. This is particularly important in the context of serious illness, where spiritual wellbeing is associated with better quality of life outcomes for patients (Whitford & Olver, 2012).

Recent investigation has shown that spiritual wellbeing and spiritual needs are independent constructs (Büssing, 2024). Therefore, absence of spiritual wellbeing does not necessarily imply the presence of spiritual need. The intensity of spiritual need can be influenced by cultural and religious issues, personality, mood states, course of disease and place of treatment (ibid). Further studies should include measurement of spiritual needs, for example using the Spiritual Needs Questionnaire (SpNQ-20) (Büssing, 2021), to ascertain the extent of spiritual need in patients with low spiritual wellbeing in order to plan appropriate spiritual care for the patient cohort who are unaware of or not experiencing spiritual need at the time of admission.

## Limitations

Several of the hospitals in this study were faith-based organisations, and our cohort reported higher levels of religiosity than the Australian average. This may have influenced the study outcomes. Further research in non-faith-based settings is recommended to confirm our findings. However, this study includes a large heterogeneous sample across a range of hospitals, including one that does not have staff chaplains, and provides useful insight into the spiritual wellbeing of Australian hospital patients.

While the FACIT-Sp-12 is a commonly used measure of spiritual wellbeing in medical research, the authors recognise that it is contaminated with indicators of mental health, and as such use of this scale could lead to misleading interpretations.

## Conclusion

Our evidence supports psychometric validity for the FACIT-Sp-11 in a heterogeneous group of Australian hospital patients. Further research is recommended to interrogate and confirm our findings and to identify the extent of spiritual need in those patients with low spiritual wellbeing.
